# Injectable radiopaque targets for cone-beam CT guided histotripsy

**DOI:** 10.1080/02656736.2026.2642172

**Published:** 2026-03-12

**Authors:** Katrina L. Falk, Michael A. Speidel, Grace M. Minesinger, Gretchen M. Foltz, Fred T. Lee, Timothy J. Ziemlewicz, Martin G. Wagner, Paul F. Laeseke

**Affiliations:** a Department of Radiology, University of Wisconsin-Madison, Clinical Sciences Center, Madison, WI, USA; b Department of Biomedical Engineering, University of Wisconsin-Madison, Madison, WI, USA; c Department of Medical Physics, University of Wisconsin-Madison, Madison, WI, USA; d Department of Urology, University of Wisconsin-Madison, Clinical Sciences Center, Madison, WI, USA

**Keywords:** Histotripsy, swine, cone-beam CT, preclinical, barium, injection

## Abstract

**Background:**

Cone-beam CT (CBCT) guided histotripsy is being developed as a supplemental image guidance technique for histotripsy. The goal of this work is to present a percutaneously injectable, radiopaque target that can be used in preclinical testing of CBCT-guided histotripsy.

**Methods:**

Livers of five *in vivo* swine were percutaneously injected with a mixture of barium paste diluted with saline or autologous blood to a concentration of 4:1. Post-injection CBCT images were used to analyze radiographic differences in injected volume; temporal stability of the injection (intensity, volume, and sphericity); and presence of image artifacts. Four injections were targeted and treated *in vivo* using a clinical histotripsy system. Injectate volume and intensity, and treatment zone size and contrast-to-noise ratio (CNR) were compared to control treatments.

**Results:**

There was no significant difference in imaged volume or intensity between the 100 or 200 μL (*p* = 0.384) injection. Over the analyzed 5–206 min, the barium-saline and barium-blood injections had similar ranges of imaged volume and intensity, both changing during the first 30 min then were stable. The image artifact was reported as none to moderate in 5/6 cases for both injections. The CNR of the treatment zones and difference in diameters (planned versus actual) were 5.9 ± 1.8, 3.7 ± 1.3, and 5.9 ± 1.0 and 8.1 ± 4.0 mm, 15.9 ± 11.4 mm, and 7.6 ± 2.2 mm for the barium-saline, barium-blood and control groups respectively. The barium-saline injectate was not visible after treatment while the barium-blood was visible but dispersed.

**Conclusion:**

Percutaneously injectable, radiopaque barium-based targets were acutely safe, targetable and treatable on CBCT and did not alter histotripsy treatment zone size *in vivo*.

## Graphical Abstract

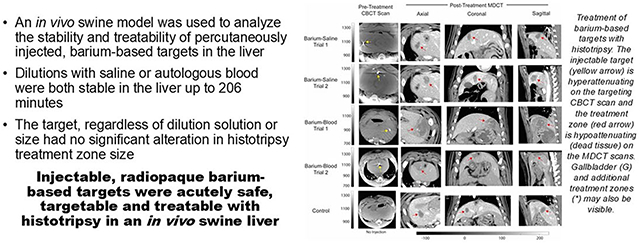

## Introduction

Histotripsy is an emerging noninvasive, non-thermal and non-ionizing focal tumor treatment that uses short high amplitude, low duty cycle focused ultrasound to destroy targeted tissue [1–5]. During cavitation histotripsy, the focused waves converge to cause a high peak negative pressure that rapidly expands and contracts nanoscale, endogenous gas bubbles [6–9]. Cavitation causes high shear stress and strain on neighboring cells to mechanically destroy tissue. The first clinical histotripsy system was cleared by the FDA in October 2023 for the treatment of liver tumors. Currently, histotripsy is guided by an integrated diagnostic ultrasound transducer, which is used to visualize and target tumors. Diagnostic ultrasound is limited by an inability to visualize beneath highly attenuating structures such as bone (ribs) and air (lung or bowel) or deep within the body. This has limited the number of patients that can be treated with histotripsy, both in recent clinical trials and in current clinical practice [10,11]. This limitation is specific to diagnostic ultrasound. The lower frequency (700 kHz versus 1–6 MHz), higher amplitude, and transducer geometry enable cavitation to be induced even in areas where the diagnostic ultrasound cannot resolve [12,13].

Cone-beam CT guided histotripsy is being developed to overcome the limitations of diagnostic ultrasound by enabling targeting of lesions that are not accessible or visible with diagnostic ultrasound [14,15]. During CBCT-guided histotripsy, the integrated robotic arm of the histotripsy system is calibrated to the C-arm through a set of mathematical transformations. The calibrated system can then automatically position the therapeutic transducer such that the focal point aligns with any target selected in a CBCT image [14–16]. Suitable *in vivo* targets are needed to further develop, characterize and translate CBCT-guided histotripsy. Placing fiducials into tissue to target is a common concept in radiation oncology, however, these are all made of metal, and the effect of metallic objects in or around the histotripsy treatment zone is unknown. Current tumor bearing animal models are limited. Small animals have been used in histotripsy immunology research, but are limited by scale and anatomical relevance [17,18]. Large animal models, such as rabbit or swine, are more anatomically relevant but are limited in options due to the inconsistent engraftment success and specific animal housing required [17,19]. Given the lack of a readily available and suitable large animal tumor model, preliminary *in vivo* work analyzed the accuracy of CBCT-guidance to target tumors using a healthy, non-tumor bearing swine model. In this study, small histotripsy treatment zones were created in livers of such swine to serve as ‘pseudotumors’ to target and treat [20]. Although results reported an accuracy of 3.8 mm, the study and accuracy assessment were limited by an inability to distinguish the centroid of the pseudotumor versus the centroid of the treatment zone created over it, especially with confounding adjacent perfusion defects from both [20].

This work presents a percutaneously injectable, x-ray visible target that can be used for preclinical testing of CBCT-guided histotripsy. An ideal target would work seamlessly in the histotripsy workflow (i.e. be injected before the coupling water bath be placed on, persist throughout the experiment, and not disrupt the histotripsy treatment zone). Therefore, the mixtures were assessed for visibility on CBCT, persistence in the liver to allow for targeting and treatment, and effects on histotripsy treatment zones.

## Materials and methods

### Animal handling and anesthesia

This study was approved by the Institutional Animal Care and Use Committee. Five healthy female swine (mean weight 57.8 kg, range 50–73 kg) were used. The swine were sedated with an intramuscular injection of tiletamine and zolazepam (Telazol, Zoetis, Kalamazoo, Michigan) and xylazine (AnaSed, Lloyd, Shenandoah, IA) and maintained with inhaled isofluorane gas for all procedures and imaging (1.5%-2.5%, Halocarbon Laboratories, River Edge, NJ). Following imaging or treatment at the end of the procedure, the animals were sacrificed with 4 mEq/kg supersaturated KCl (EMD milipore Corp., Burlington, MA) while under anesthesia. Conventional ventilation was performed with no respiratory motion mitigation.

### Experimental design

**Figure 1.**
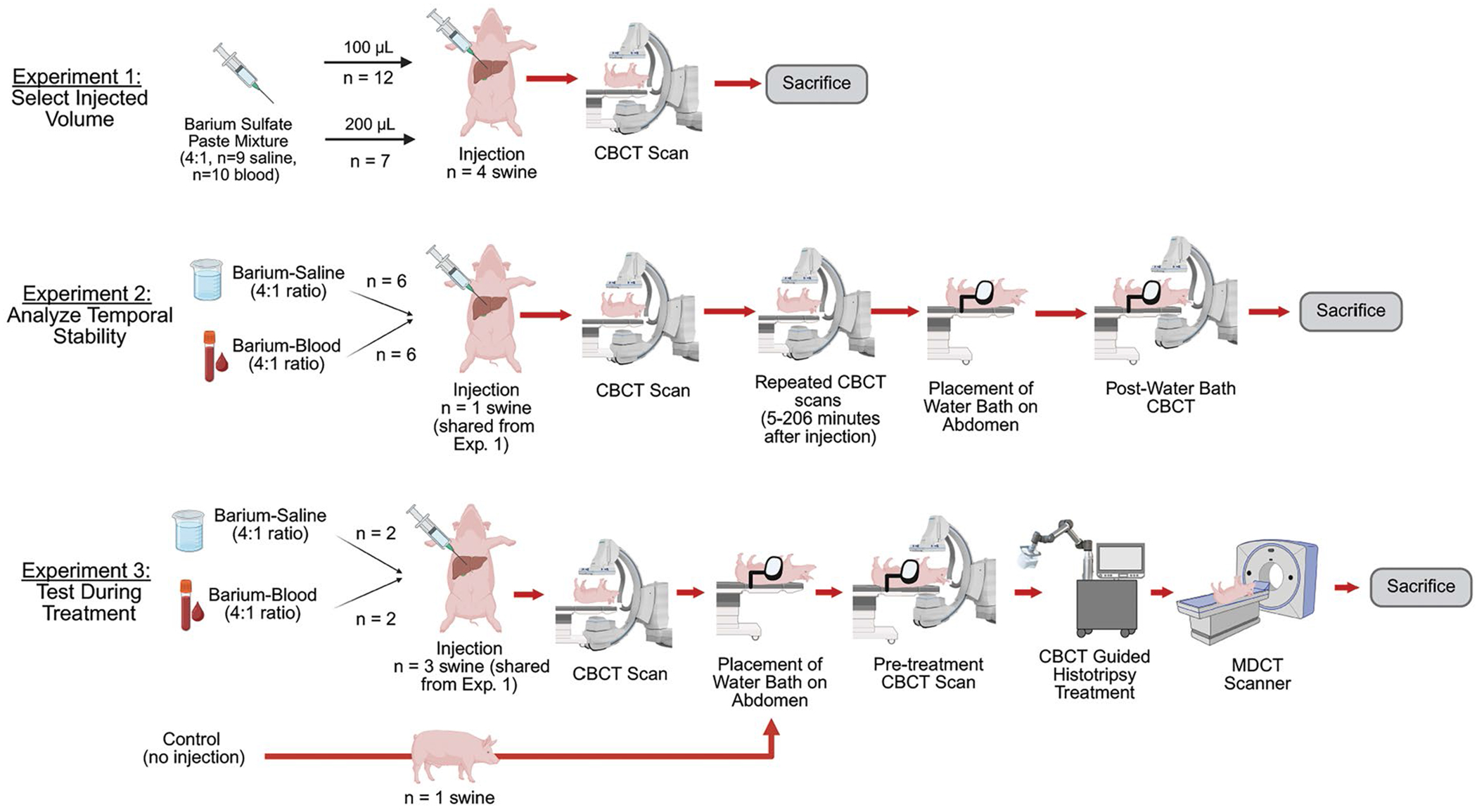
Diagram of the experimental design. Experiment 1 compared different injection volumes. Experiment 2 compared the persistence of the injections over time and Experiment 3 analyzed if the histotripsy treatment zone size was affected by treating on an injection. Figure made with BioRender [21].

A mixture of barium paste (E-Z-Paste, Barium Sulfate Esophageal Cream, 60% w/w, Bracco Diagnostics Inc, Monroe Twp, NJ), was diluted with saline or autologous blood to a concentration of 4:1. Autologous blood was collected via an auricular vein catheter immediately before mixing. The mixture was loaded into a 1 ml Luer lock syringe and connected to a 20-gauge x 15 cm Chiba biopsy needle (Argon Medical Devices, Athens, Tx). The needle was purged of air and inserted percutaneously into the liver of the supine swine under ultrasound guidance, avoiding vessels and critical structures. Three experiments were conducted (Figure 1) and post-injection imaging was done using one of three clinical mobile or fixed CBCT imaging systems: Artis Zee (Siemens Healthineers, Forcheim, Germany), CIOS (Siemens Healthineers, Forcheim Germany), and OEC (GE Healthcare, Salt Lake City, UT). All CBCT images were reconstructed with the vendor’s metal artifact reduction kernel.

In Experiment 1, mixtures of barium-saline and barium-blood were injected at volumes of 100 μL (*n* = 12) or 200 μL (*n* = 7) into independent areas of the liver in four swine. A post-injection CBCT scan (2 animals with the Artis Zee, 1 with the CIOS and 1 with the OEC) was obtained, and the volume and intensity of injectate were analyzed (information in analysis section) to select the injection volume with optimal visibility.

In Experiment 2, mixtures of barium-saline and barium-blood were injected into one animal in independent areas of the liver, and repeated scanning was performed to evaluate temporal stability of the injectate. All injections were 100 μL, as determined from Experiment 1. All CBCT scans in this experiment were acquired with the fixed C-arm. CBCT scans were acquired directly after and then 5–206 min post-injection. The mixtures were injected in an alternating order to balance the number of consecutive scans for each mixture. After the last scan, a clinical histotripsy coupling device containing ~14 L of water (used to acoustically couple the therapeutic energy during treatments) was placed on the abdomen of the animal and a CBCT scan was acquired. The injectate intensity, volume, and sphericity were calculated (information in analysis section) from the scan obtained with the coupling water in place.

In Experiment 3, three swine were injected (100–200 μL) with either barium-saline (*n* = 2) or barium-blood (*n* = 2) into independent areas of livers to test as a target for histotripsy treatment. One additional swine was used as a control where a treatment was performed in healthy liver (no injection). A CBCT scan (1 animal with the Artis Zee, 1 with CIOS and 1 with OEC) was obtained after injection and a water bath with 12–14 L of degassed water was placed on the abdomen after the typical clinical preparation of the abdominal surface (hair removal and coupling oil). A pre-treatment CBCT scan was acquired and used for targeting. Calibration of the histotripsy system to the fixed C-arm was performed using previously described techniques [14–16,22]. The injectate was selected in the 3D volume and the therapy transducer automatically focused on the target. A clinical histotripsy system and research transducer were used (700 kHz, 56 element, f number 0.97/0.64 for the short/long axis, HistoSonics, Inc, Plymouth, MN). Spherical treatment volumes (1.5–2.5 cm) were created centered on the targets with the animal under conventional mechanical ventilation. Post-treatment imaging was performed with a clinical multi-detector CT scanner (64 slice, 40 mm max collimation, Discovery CT750 HD, GE Healthcare, Waukesha, WI) and the animal was subsequently euthanized. The injectate intensity and volume were calculated (information in analysis section) pre- and post-treatment.

### Analysis

Qualitative and quantitative assessment of the injectates varied in each experiment. Experiment 1 analyzed imaging volume and intensity of different volumes of injectate. Experiment 2 analyzed successful establishment and temporal stability (injectate volume, intensity, and sphericity). Experiment 3 tested the injectate as a target for histotripsy treatment (size and contrast-to-noise ratio (CNR) of the treatment zone). For all experiments, all CBCT image values were converted to Hounsfield Units (HU) using Equation 1,

(1) $$i_{\mathrm{HU}}=a_{\mathrm{HU}}+\left(i_{\mathrm{CBCT}}-a_{\mathrm{CBCT}}\right)\left(\frac{b_{\mathrm{HU}}-a_{\mathrm{HU}}}{b_{\mathrm{CBCT}}-a_{\mathrm{CBCT}}}\right)$$

where *i_CBCT_* is the value at a voxel in the CBCT scan, *a_HU_* is the HU value of air, *b_HU_* is the HU of bone, *a_CBCT_* is the intensity value of air on CBCT, and *b_CBCT_* is the intensity value of bone on CBCT. This equation is a linear fit to convert CBCT intensities to HU. The reference HU values were measured via a MDCT scan of a swine of similar size (kV 120). All image post-processing was done with MATLAB (2021a) and all averages are reported as a mean ± standard deviation.

Experiment 1: The density of each injection was measured by dividing the mass (measured with a scale, Adventurer Pro AV114C, Ohaus Corp., Parsippany, NJ) by the volume and computing the average of 6 biological replicates. Successful establishment of each injection was qualitatively determined by visualizing the injectate on the CBCT scan immediately after injection. To measure the image volume and intensity, a bounding box was created around each injection and a Chan-Vese segmentation algorithm (activecontour) was used to automatically segment the injectate [23]. The largest island of voxels with the 99^th^ percentile of HU values was used as the seed volume for the algorithm. The output of the segmentation was used to calculate the image volume (μL) and intensity (average HU within the mask) of the injectate (regionprops3). A Shapiro Wilk test was used to test for non-normality and a Mann-Whitney U test was used to find statistical significance between injected volumes (α = 0.05).

Experiment 2: The imaged volume and intensity were measured as above. In addition to the volume, the surface area (mm^2^) of the injectate was measured (regionprops3) and used to calculate the sphericity (measure of how a shape deviates from a sphere where 1 represents a perfect sphere). To describe the temporal changes of the imaged volume and intensity during the stable period, a linear fit was done to remove the noise and used to evaluate the absolute and percent change. A linear regression analysis was performed to assess the relationship between the volume and intensity of each injectate. A simple linear model was fitted using MATLAB (fitlm) and the coefficient of determination (R^2^) was extracted to evaluate the strength and significance of the association.

The artifact and visibility of the injectate was rated by four board certified radiologists with combined 77 years of experience via a five-point Likert scale: 1 = no artifact, no impact on visibility of adjacent anatomy, 2 = minimal artifact, minimal impact on visibility of adjacent tissue, 3 = moderate artifact, some impact on visibility of adjacent tissue, 4 = moderate-severe artifact, major impact on visibility of adjacent anatomy, 5 = severe artifact, significantly impacting visibility of adjacent and distant anatomy. To mimic the clinical targeting scenario and to minimize confounding effects of motion and window leveling, one scan of all the injections with the water bath in place was used to assess injectate artifacts. A Fleiss’ Kappa was used to determine the level of agreement among the four reviewers.

Experiment 3: To test if the target affected the treatment zone, the CNR was calculated from the post-treatment, contrast-enhanced MDCT image using Equation 2,

(2) $$\mathrm{CNR}=\frac{i_{T}-i_{L}}{n_{L}}$$

where *i_T_* is the intensity (HU) of the treatment zone, *i_L_* is the intensity (HU) of the background liver, and *n_L_* is the standard deviation of the intensity in the background liver. Measurements were completed by one user by manually drawing a region of interest (ROIs) in the treatment zone on the MDCT scan via the picture archiving and communication system (PACS) (Change Healthcare Radiology Solution, Nashville, TN). Three different ROI measurements were used to compute the final CNR. Dimensions were compared to the planned size for the barium-blood, barium-saline and control groups. In addition, diameters of the histotripsy treatment zones were manually measured on 2D slices of MDCT images in anteroposterior, craniocaudal, and lateral dimensions and compared to the planned treatment zone size. The average percentage error (%) between the measured and planned dimensions was calculated and compared to control. The volume and intensity of injectate were measured on the pre- and post-histotripsy treatment using the same algorithm as previously mentioned.

## Results

### Experiment 1

**Figure 2.**
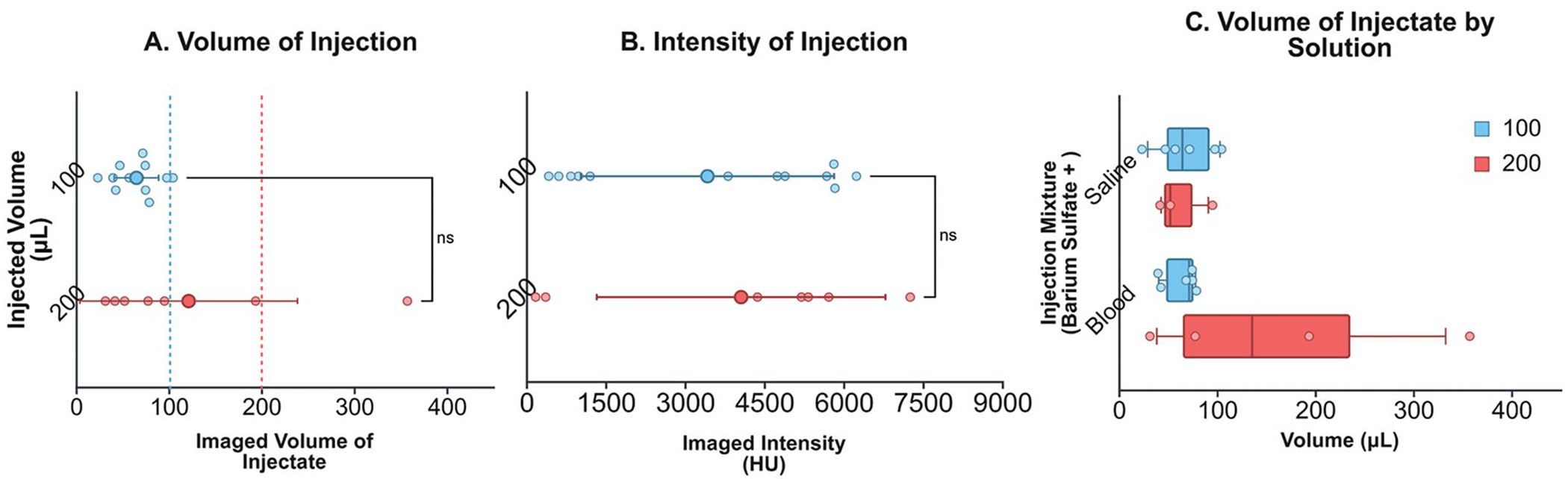
Volume and image intensity of barium-based injections on the cone-beam CT (CBCT) image immediately post injection. A) Volume comparison of barium-based (both blood and saline) injections of 100 and 200 μL. B) Intensity comparison of barium-based (both blood and saline) injections of 100 and 200 μL. C) Individual analysis of volume of barium-saline and barium-blood injections from CBCT images immediately post injection. Vertical dotted lines represent the initial injection quantity. ns = no statistically significant difference. Bars represent the 5^th^ and 95^th^ percentiles. Figure made with BioRender [21].

The density of the barium-saline and barium-blood mixtures were 17.85 ± 2.1 g/mL and 18.26 ± 1.0 g/mL, respectively. 19/19 injections were successfully established in the swine liver. Figure 2A presents the imaged volume versus injected volume. For the 12 injections of 100 μL, the imaged volume on CBCT was 65.1 ± 23.9 μL and for the 7 injections of 175–200 μL, it was 121.4 +− 117.4 μL. Figure 2B presents the imaged intensity versus injected volume. The average image intensities were similar for the two injected volumes, 3423 ± 2395 HU for 100 μL and 4060 ± 2731 HU for 200 μL. The Shapiro–Wilk test indicated that the data significantly deviated from a normal distribution, W(12) = 0.82, *p* = .02 so a Mann Whitney U test was conducted and failed to show significant differences in imaged volume or intensity between the 100 μL (*n* = 12) and 200 μL group (*n* = 7), *U* = 34, *p* = 0.536 and *U* = 41, *p* = 0.967, respectively. Figure 2C breaks down the injections by solution (saline vs. blood). The barium-blood mixture injected at 200 μL had the largest range of imaged volumes (31.6–357.4 μL). Since the two injected volumes provided similar average image volumes and average intensities, the smaller injected volume (100 μL) was selected for Experiment 2 and 3.

### Experiment 2

**Figure 3.**
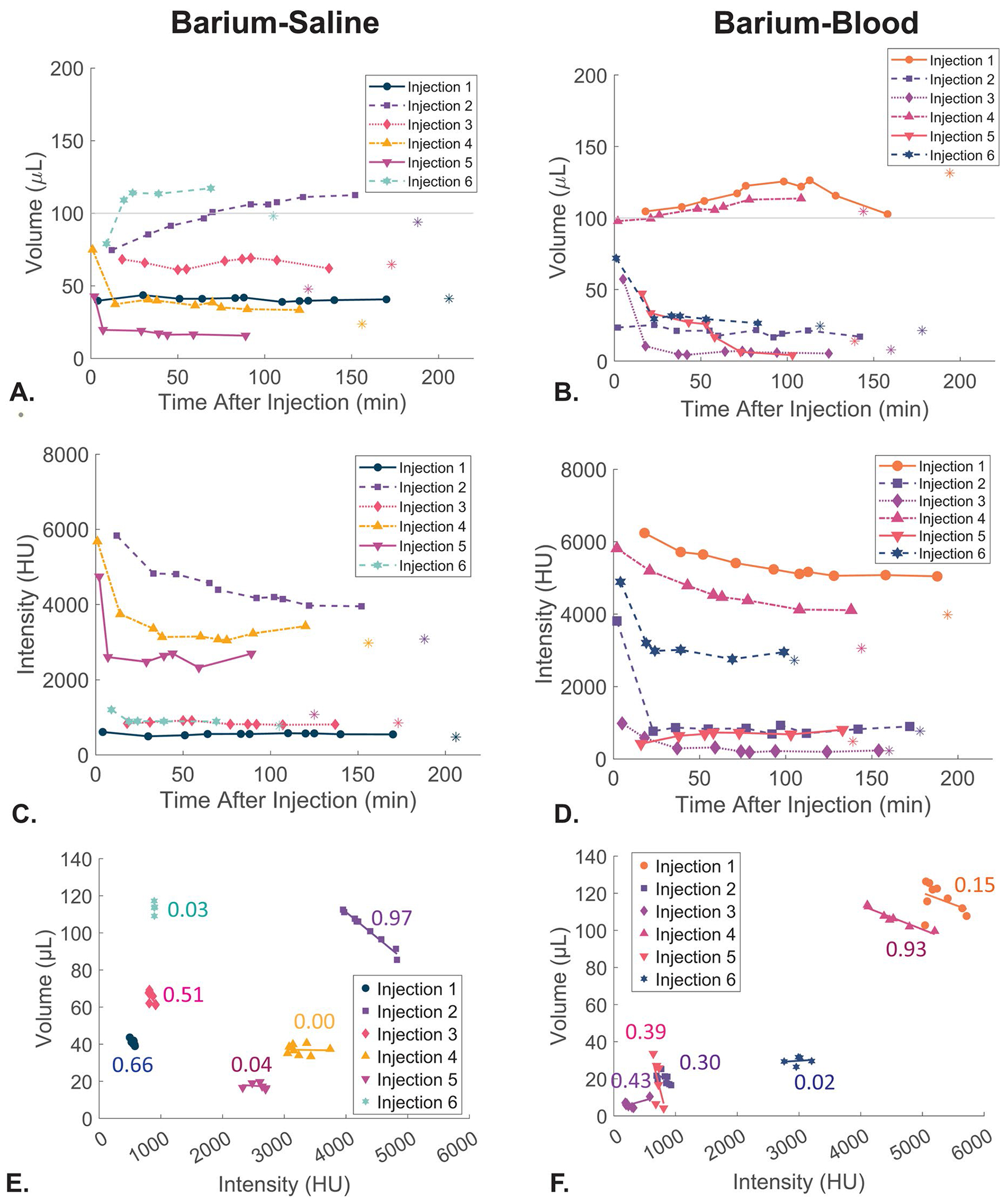
Volume (A&B), intensity (C&D), of the 12 injectates (barium-saline (left) and barium-blood (right)) over time. Values were measured from the repeated post-injection cone-beam CT images with intensity values converted to HU. Some measurements start at timepoints greater than zero due to motion artifact that required omission of scans. The horizontal line in A&B represents the injected volume. Graphs E and F represent the correlation between volume and intensity for each injectate during the stability period. The line of best fit and R^2^ value for each injection is shown. Two injections had a strong negative correlation.

6/6 barium-saline and 6/6 barium-blood mixtures (100 μL) were successfully established and persisted on repeated CBCT scans (up to 97–206 min post-injection). Figure 3 presents the temporal stability of the injected targets. The two groups resulted in an overlapping range of imaged volumes and intensities averaged across the six injections: 21.1–106.6 μL and 557.7–4486.9 HU for barium-saline and 12.0–115.6 μL and 359.1–5370.6 HU for barium-blood. For both injection types, there was a period of volume and intensity change for the first 30 min followed by a period of stability. Once stabilized, there was on average a 3.8% decrease to the volume and 2.5% change to the intensity for the barium-saline group and 16.8% and 7% for the barium-blood group, respectively. Interestingly, both the 16.8% and 3.8% overall change equated to *a* ~9 μL absolute change, with one outlier skewing the percent in the barium-blood group. The linear regression analysis (Figure 3E and 3F) was performed during the stability period. It showed a strong negative relationship between volume and intensity in injection 2 (R^2^ = 0.97) in the barium-saline group and injection 4 (R^2^ = 0.93) in the barium-blood group. The other injections were stable across the timepoint and lacked a positive or negative relationship between volume and intensity of the injectate (R^2^ values found on the graphs in Figure 3E and 3F). Table 1 shows the location of each injection within the liver and distance to the nearest liver capsule. The two injectates that had a negative correlation (barium-saline injection 2 and barium-blood injection 4) were both located furthest from the capsule, suggesting they were most central in the liver (Table 1).

The imaged volume after water bath placement (labeled as the asterisks in Figure 3) decreased by an average of 2.0 ± 19.1 μL in the barium-saline injections and increased by an average of 5.7 ± 12.9 μL in the barium-blood injections after the water bath. Although adding water decreased image quality, all injections were easily visible on the CBCT with an intensity greater than 186.4 HU. Measurements of injectate sphericity were consistent over time with an average change of −0.4% ± 13% across the 12 injections.

**Figure 4.**
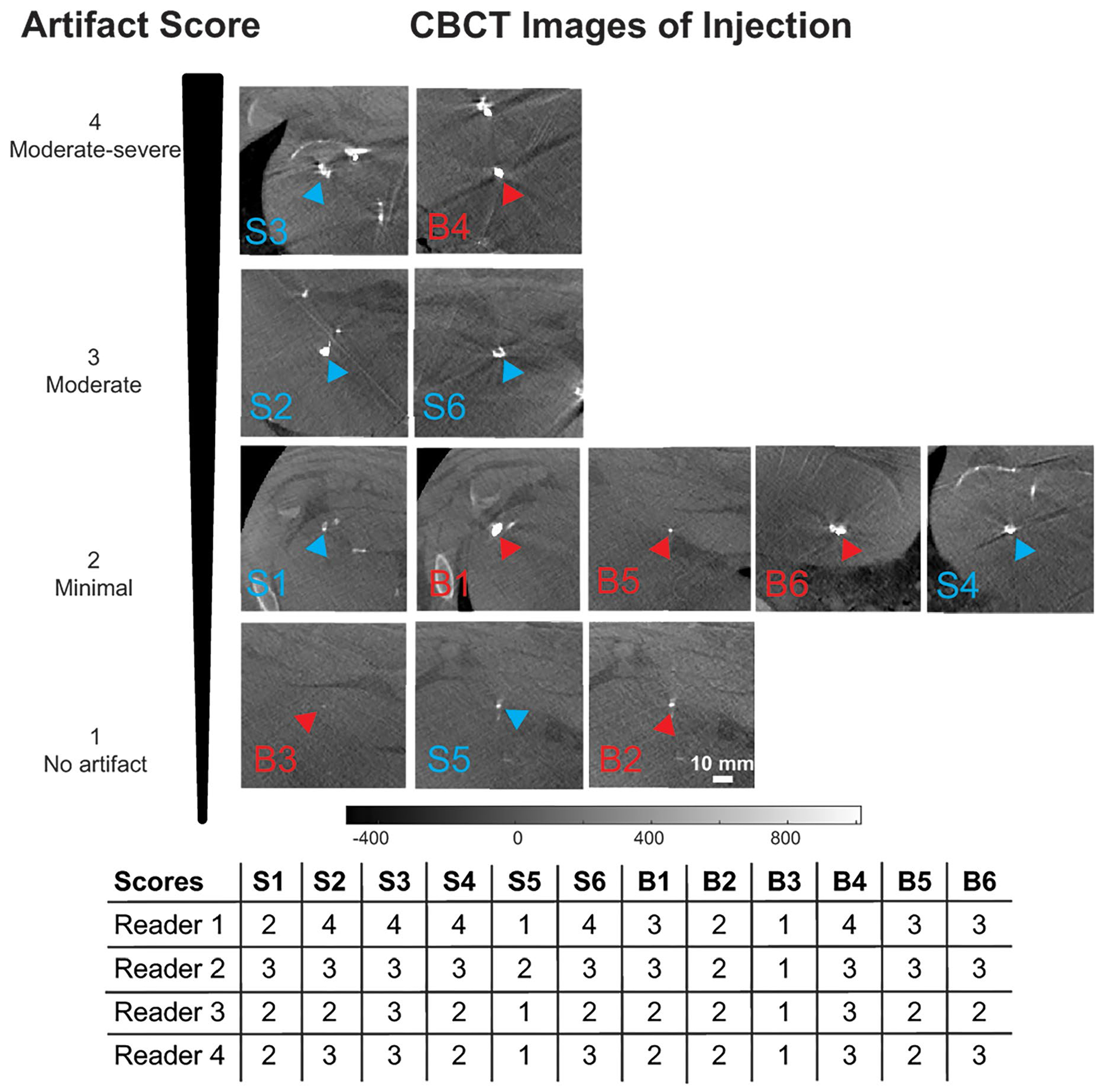
Artifact scores for 12 percutaneous injections (S = barium-saline, B = barium-blood) rated by four blinded radiologists. Injections were rated from a CBCT image with the water bath, on a Likert Scale: 1 = no artifact, no impact on visibility of adjacent anatomy, 2 = minimal artifact, minimal impact on visibility of adjacent tissue, 3 = moderate artifact, some impact on visibility of adjacent tissue, 4 = moderate-severe artifact, major impact on visibility of adjacent anatomy, 5 = severe artifact, significantly impacting visibility of adjacent and distant anatomy. All scores were below 4. Arrowheads outlined in blue and red point to the barium-saline and barium-blood injections, respectively. A scale bar represents HU values converted from CBCT intensities.

CBCT images of the barium-based targets immediately post-injection were analyzed for an artifact score by four radiologists blinded to the injection group. Figure 4 shows the barium-based injections and corresponding artifact score. The mean artifact score for the 6 barium-saline injections was 2.58 ± 0.74 and was 2.29 ± 0.79 for the 6 barium-blood injections. There was an equal distribution of cases (5/6) with artifact scores less than or equal to 3 for both injection groups. Fleiss’ Kappa indicated slight agreement (ĸ = 0.18).

### Experiment 3

**Figure 5.**
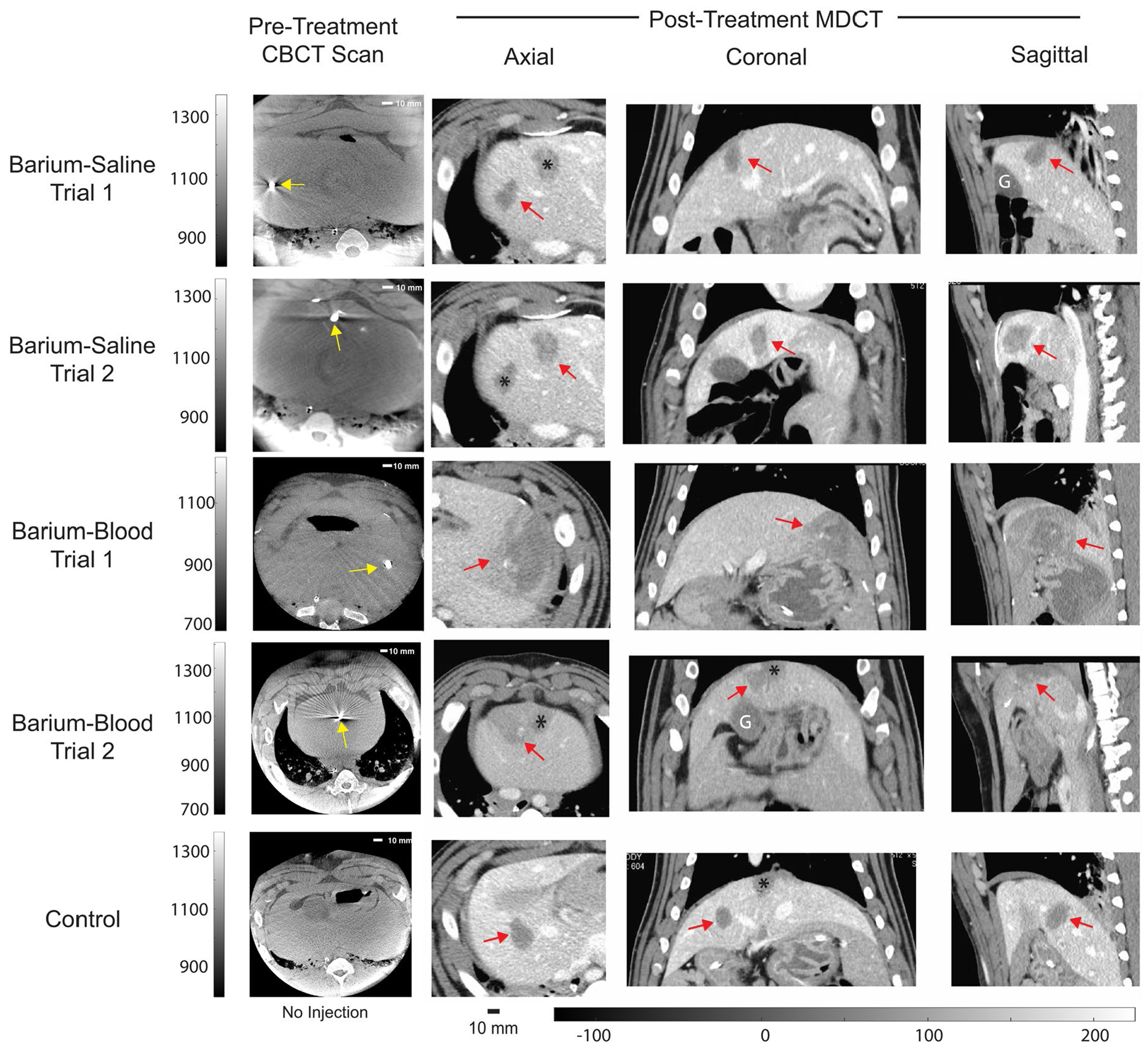
Visualization of the effect of treating barium-based targets. Pretreatment cone-beam CTs (CBCT) illustrate each percutaneously injected target (yellow arrow). After histotripsy, the treatment zone (red arrows) can be seen on the axial, coronal and sagittal views portal venous phase MDCT with contrast. No residual injectate was seen in the barium-saline group, but some injectate remained in the barium-blood group after treatment. Peripheral perfusion defects of healthy tissue are present adjacent to the treatment zone in the barium-blood group due to location of the treatment zone in the liver. Additional treatment zones that happen to be included in the field of view are marked with an asterisk. The gallbladder is labeled with G. Scale bars for the CBCT intensity values of each pretreatment CBCT and HU values for each CT are present (all CTs share one scale bar).

CNR of the treatment zone was on average, 5.9 ± 1.8 for the barium-saline mixture, 3.7 ± 1.3 for the barium-blood mixture, and 5.9 ± 1.0 for the control. Figure 5 shows the pretreatment CBCT (with water bath) used to target the barium mixture and the corresponding histotripsy treatment zones on the post-treatment MDCT. The barium-blood Trial 1 had an unrelated system error which led to retargeting and restarting the treatment. The average measured treatment zone diameters are listed in Table 2. Across all anatomical planes, the average difference between planned and actual treatment diameters was 8.1 ± 4.0 mm for the barium-saline, 15.9 ± 11.4 mm for barium-blood, and 7.6 ± 2.2 mm for the control treatment. Table 3 shows the volume and intensity of the injectate before and after treatment. After treatment, the barium-blood injectate dispersed, increasing in volume by 2.8 times and 12.9 times and decreasing in intensity by 37.8 times and 27.0 times in Trial 1 and Trial 2, respectively. Both barium-saline injections fully dispersed and were no longer visible.

## Discussion

Cone beam CT (CBCT) is being developed as an imaging modality to target tumors for histotripsy treatment. Previous preclinical work on the accuracy of CBCT guided histotripsy used healthy *in vivo* swine which required first creating a small histotripsy treatment zone to serve as a pseudotumor target and then overtreating with a larger histotripsy treatment zone [20]. Using histotripsy to create both the target and treatment zone confounded and limited targeting accuracy measurements due to overlapping zones and perfusion changes to adjacent parenchyma. A porcine liver tumor model would enable more accurate evaluations of CBCT guided histotripsy, however, there is only one tumor model, and it is cost-prohibitive and not yet widely available. Placing fiducials into tissue to target is a common concept in radiation oncology, however, these are all made of metal, and the effect of metallic objects in or around the histotripsy treatment zone is unknown. Therefore, to utilize the healthy porcine swine model for CBCT guided histotripsy research, a percutaneously injectable, x-ray visible target was developed and evaluated for its visibility, persistence over time, and effect on histotripsy treatment zone visibility.

The targets were created out of barium sulfate paste, which is commonly used in radiographic swallow studies. The barium paste was diluted with either saline or autologous blood, to achieve a viscosity that could be deployed through a needle while also maximize chance of retention. From Experiment 1, the two quantities of the injectate (100 and 200 μL) resulted in final imaged volumes of approximately 100 μL or less, regardless of the injected amount. Moreover, the range of image intensities was similar for the two injected volumes. Since the larger volume did not yield substantial increases in imaged volume or intensity, the 100 μL volume was selected for subsequent experiments.

In Experiment 2 (100 μL injections of barium mixed with either saline or autologous blood), there was an initial period of change (<30 min) followed by a prolonged period of stability of the imaged volume and intensity over the subsequent 67–176 min. This is important, as the animal prep (shaving of the abdomen, oiling the skin, and placement of the water bath) takes around 20–30 min so histotripsy targeting and treatment during a preclinical study would occur during the target stability period. The initial changes in image intensity and volume can result from either flow into adjacent vessels or peritoneum or diffusion into adjacent parenchyma. In the injections that were ≤ 20 mm from a capsule (i.e. peripheral injections), there was no correlation between volume and intensity, suggesting their relationship was consistent during the stability period. In the two injections that were central in the liver (barium-blood #4, 22.2 mm and barium-saline #2, 33.5 mm from the posterior capsule) had a strong negative correlation between volume and intensity suggesting dispersion into adjacent parenchyma. The findings Experiment 1 and 2 suggest the swine liver may have a limited capacity to store a local volume of injectate. After a histotripsy coupling water bath was placed on the abdomen, the injections had *a* < 6% overall change in volume. Although the addition of the water bath degrades image quality due to increases in scatter, attenuation and beam hardening [24], every injection remained visible on the CBCT scan (Figure 4). Radiologists determined the visibility and persistence of the injectate at 97–206 min post-injection was adequate for CBCT based targeting.

From Experiment 3, the greatest change in diameter of the histotripsy treatment zone was in the cranial-caudal dimension due to respiratory motion, consistent with previously reported work on *in vivo* histotripsy in healthy swine [25,26]. Trial 1 of treating on the barium-blood mixture had an unexpected system error during treatment which led to retargeting and restarting the treatment which could explain the large size. Peripheral perfusion defects of healthy tissue were also present adjacent to the treatment zone in the barium-blood group due to location of the treatment zone in the liver and treatment over a vessel. This is a common imaging finding of histotripsy treatments in the liver and resolves. In the post-treatment images, there was a different amount of detectable residual injectate within the treatment zones. The barium-blood mixture left some residual injectate in the treatment zone, likely because of the blood coagulation hindering dissipation. However, there was no evidence of the barium-saline injectate after treatment, suggesting that the user can tailor the presence of the target post-treatment by mixing with either saline or blood. Since they both were shown to act similarly upon injection, a possible situation where this may be useful is if multiple treatments are done in the same liver, a user can use one barium-saline mixture and one barium-blood mixture to easily delineate which treatment is which.

There are several limitations of this study. Although the needle and syringe were prepared to deliver the precise amount of injectate, it is likely that the exact amounts varied across injections. Diagnostic ultrasound was used to avoid vessels and fissures; however, it is possible that some injectate was lost in the peritoneum via the insertion tract or into the vasculature. An example of this can be seen in the anterior liver of the barium-blood injection in Figure 5. The sagittal view shows increased attenuation in a distal vessel, suggesting extravasation of the injectate into the vasculature. This did not affect targeting, nor did it impact the animal acutely, but the effect of this injection technique on chronic animals is unknown and requires further study. This study tests only injections with barium sulfate paste and does not test other materials that are CT attenuating, like implantable fiducials. This work also does not include any histologic analysis on the treatment zone or data on long term safety of using this injectate in a chronic swine study. Future work should include this. Future work could also feasibly include analysis of barium dilution to mimic a hyperattenuating liver lesion *in vivo*.

## Conclusion

This work presents a percutaneously injectable, x-ray visible target for *in vivo*, acute abdominal histotripsy studies. The injection is made from mixing barium sulfate paste with either saline or blood. By analyzing successful establishment, volume, intensity, and artifact of these two injection types, as well as effect on the histotripsy treatment zone, results suggest that using both barium-saline and barium-blood are feasible injectates to target using CBCT guidance. The barium-saline mixture disappeared after treatment while the barium-blood mixture had some remain, suggesting that visibility post-treatment can be tailored for each study. Both injection mixtures were easily visible with CBCT, persisted in the liver for hours to allow for same day targeting and treatment, were acutely safe for the animals and did not disrupt the treatment zone size. This study presents percutaneously injected, barium-based targets for future facilitation of CBCT guided histotripsy preclinical research.

## Figures and Tables

**Table 1.** Injectate locations within the liver.

	Injection	Location within liver	Capsule it was closest to on a sagittal image	Distance from capsule (mm)
Barium-Saline	1	Right Medial	Anterior	6.1
	2	Right Lateral	Posterior	33.5
	3	Right Medial	Cranial	12.6
	4	Right Medial	Cranial	20.0
	5	Right Medial	Anterior	14.1
	6	Left Medial	Anterior	15.9
Barium-Blood	1	Right Medial	Anterior	7.3
	2	Right Medial	Anterior	9.9
	3	Left Medial	Anterior	11.2
	4	Left Medial	Posterior	22.6
	5	Left Medial	Anterior	9.4
	6	Left Lateral	Cranial	12.0

**Table 2.** Histotripsy treatment zone measurements from the portal venous phase MDCT with contrast.

	Planned Size (mm)	Anteroposterior (mm)	Craniocaudal (mm)	Lateral (mm)	Average absolute difference (measured – planned) (mm)
Barium-Saline Trial 1	15	24.5	26.8	16.8	7.7 ± 5.2
Barium-Saline Trial 2	15	25.4	25.5	19.4	8.4 ± 3.5
Barium-Blood Trial 1	25	57.1	53.1	34.3	23.2 ± 12.2
Barium-Blood Trial 2	25	38.2	29.7	33.1	8.7 ± 4.3
Control (no barium)	15	24.4	23.3	20.2	7.6 ± 2.2

**Table 3.** Barium injectate properties before and after treatment.

	Volume Before Treatment (μL)	Volume After Treatment (μL)	Intensity Before Treatment (HU)	Intensity After Treatment (HU)
Barium-Saline Trial 1	52.1	Not visible	4608.4	Not visible
Barium-Saline Trial 2	73.6	Not visible	3545.9	Not visible
Barium-Blood Trial 1	149.3	419.0	5433.5	143.5
Barium-Blood Trial 2	83.5	1084.6	4833.1	178.7

## Data Availability

The datasets used and/or analyzed during the current study are available from the corresponding author on reasonable request.

## References

[1] Xu Z, Ludomirsky A, Eun LY, Controlled ultrasound tissue erosion. IEEE Trans Ultrason Ferroelectr Freq Control. 2004;51(6):726–736. doi: 10.1109/tuffc.2004.1304271. 15244286 PMC2669757

[2] Khokhlova VA, Fowlkes JB, Roberts WW, Histotripsy methods in mechanical disintegration of tissue: towards clinical applications. Int J Hyperthermia. 2015;31(2):145–162. doi: 10.3109/02656736.2015.1007538. 25707817 PMC4448968

[3] Vlaisavljevich E, Maxwell A, Mancia L, Visualizing the histotripsy process: bubble cloud–cancer cell interactions in a tissue-mimicking environment. Ultrasound Med Biol. 2016;42(10):2466–2477. doi: 10.1016/j.ultras-medbio.2016.05.018. 27401956 PMC5010997

[4] Vlaisavljevich E, Kim Y, Owens G, Effects of tissue mechanical properties on susceptibility to histotripsy-induced tissue damage. Phys Med Biol. 2014;59(2):253–270. doi: 10.1088/0031-9155/59/2/253. 24351722 PMC4888779

[5] Xu Z, Khokhlova TD, Cho CS, Histotripsy: a method for mechanical tissue ablation with ultrasound. Annu Rev Biomed Eng. 2024;26(1):141–167. doi: 10.1146/annurev-bioeng-073123-022334. 38346277 PMC11837764

[6] Williams RP, Simon JC, Khokhlova VA, The histotripsy spectrum: differences and similarities in techniques and instrumentation. Int J Hyperthermia. 2023;40(1):2233720. doi: 10.1080/02656736.2023.2233720. 37460101 PMC10479943

[7] Bader KB, Vlaisavljevich E, Maxwell AD. For Whom the bubble grows: physical principles of bubble nucleation and dynamics in histotripsy ultrasound therapy. Ultrasound Med Biol. 2019;45(5):1056–1080. doi: 10.1016/J.ULTRASMEDBIO.2018.10.035. 30922619 PMC6524960

[8] Vlaisavljevich E, Maxwell A, Warnez M, Histotripsy-induced cavitation cloud initiation thresholds in tissues of different mechanical properties. IEEE Trans Ultrason Ferroelectr Freq Control. 2014;61(2):341–352. doi: 10.1109/TUFFC.2014.6722618. 24474139 PMC4158820

[9] Xu Z, Hall TL, Vlaisavljevich E, Histotripsy: the first noninvasive, non-ionizing, non-thermal ablation technique based on ultrasound. Int J Hyperthermia. 2021;38(1):561–575. doi: 10.1080/02656736.2021.1905189/FORMAT/EPUB. 33827375 PMC9404673

[10] Vidal-Jove J, Serres X, Vlaisavljevich E, First-in-man histotripsy of hepatic tumors: the THERESA trial, a feasibility study. Int J Hyperthermia. 2022;39(1):1115–1123. doi: 10.1080/02656736.2022.2112309. 36002243

[11] Ziemlewicz TJ, Critchfield JJ, Mendiratta-Lala M, The #HOPE4LIVER single-arm pivotal trial for histotripsy of primary and metastatic liver tumors 1-year update of clinical outcomes. Ann Surg. 2025;282(6):908–916. doi: 10.1097/SLA.0000000000006720. 40201962 PMC12594125

[12] Knott EA, Longo KC, Vlaisavljevich E, Transcostal histotripsy ablation in an in vivo acute hepatic porcine model. Cardiovasc Intervent Radiol. 2021;44(10):1643–1650. doi: 10.1007/s00270-021-02914-1. 34244841

[13] Sukovich JR, Xu Z, Kim Y, Targeted lesion generation through the skull without aberration correction using histotripsy. IEEE Trans Ultrason Ferroelectr Freq Control. 2016;63(5):671–682. doi: 10.1109/TUFFC.2016.2532504. 26890732 PMC7371448

[14] Wagner MG, Periyasamy S, Kutlu AZ, An X-ray C-arm guided automatic targeting system for histotripsy. IEEE Trans Biomed Eng. 2023;70(2):592–602. doi: 10.1109/TBME.2022.3198600. 35984807 PMC9929026

[15] Minesinger GM, Laeseke PF, Falk KL, Accuracy and reproducibility of a single-pose image-to-robot registration method for mobile C-arm cone-beam CT guided histotripsy. J Appl Clin Med Phys. 2025;26:e70132. doi: 10.1002/acm2.70132. 40450384 PMC12256563

[16] Falk KL, Laeseke PF, Minesinger GM, Calibration correction to improve registration during cone-beam CT guided histotripsy. Med Phys. 2025;52(5):3216–3227. doi: 10.1002/mp.17644. 39865624 PMC12059542

[17] Hendricks-Wenger A, Arnold L, Gannon J, Histotripsy ablation in preclinical animal models of cancer and spontaneous tumors in veterinary patients: a review. IEEE Trans Ultrason Ferroelectr Freq Control. 2022;69(1):5–26. doi: 10.1109/TUFFC.2021.3110083. 34478363 PMC9284566

[18] Falk KL, Laeseke PF, Kisting MA, Clinical translation of abdominal histotripsy: a review of preclinical studies in large animal models. Int J Hyperthermia. 2023;40(1):2272065. doi: 10.1080/02656736.2023.2272065. 37875279 PMC10629829

[19] Pascale F, Pelage JP, Wassef M, Rabbit VX2 liver tumor model: a review of clinical, biology, histology, and tumor microenvironment characteristics. Front Oncol. 2022;12:871829. doi: 10.3389/fonc.2022.871829. 35619923 PMC9128410

[20] Wagner MG, Minesinger GM, Falk KL, Evaluation of targeting accuracy of cone beam CT guided histotripsy in an in vivo porcine model. Int J Hyperthermia. 2025;42(1):2455138. doi: 10.1080/02656736.2025.2455138. 39842812 PMC11784921

[21] Falk K Created in BioRender; 2025. https://BioRender.com/409nuc9.

[22] Wagner MG, Laeseke PF, Kutlu A, A histotripsy targeting approach using a mobile C-arm. Proc SPIE 12463, Med Imaging 2023 Phys Med Imaging 124630O:25; 2023. doi: 10.1117/12.2654002.

[23] Chan TF, Vese LA. Active contours without edges. IEEE Trans Image Process. 2001;10(2):266–277. doi: 10.1109/83.902291. 18249617

[24] Minesinger G, Wagner M, Kutlu A, Mobile C-arm–guided histotripsy: Assessment of image quality in an in vivo porcine model. J Vasc Interv Radiol. 2023;34(3):S25–S26. doi: 10.1016/j.jvir.2022.12.095.

[25] Longo KC, Zlevor AM, Laeseke PF, Histotripsy ablations in a porcine liver model: feasibility of respiratory motion compensation by alteration of the ablation zone prescription shape. Cardiovasc Intervent Radiol. 2020;43(11):1695–1701. doi: 10.1007/s00270-020-02582-7. 32676957 PMC8543737

[26] Winterholler JE, Kisting MA, Falk KL, Hepatic histotripsy: jet ventilation decreases the effect of respiratory motion in a porcine liver model. Cardiovasc Intervent Radiol. 2025;48(8):1164–1173. doi: 10.1007/s00270-025-04060-4. 40468037 PMC12325549

